# Orthodontic Management of the Systemically Healthy Gravid Patient: An Overview and Proposed Clinical Framework

**DOI:** 10.3390/dj14080467

**Published:** 2026-08-02

**Authors:** Riccardo Favero, Sadaf Suleman, Andrea Volpato, Christian Bacci, Vittorio Favero

**Affiliations:** 1 Department of Neuroscience, University of Padua, Via Giustiniani, 1, 35100 Padua, Italy; riccardo.favero@unipd.it (R.F.); sadaf.suleman@studenti.unipd.it (S.S.); christian.bacci@unipd.it (C.B.); 2 Department of Medicine and Surgery, University of Padua, Via Giustiniani, 1, 35100 Padua, Italy; vittorio.favero@unipd.it

**Keywords:** pregnancy, orthodontics, orthodontic-treatment, pregnant-patient, oral-health, gravid-patient

## Abstract

Orthodontic treatment can be safely performed during pregnancy when tailored to the patient’s hormonal, physiological, psychological, and oral health changes. The study aims to establish a safe, trimester-specific orthodontic practical framework that minimizes maternal–foetal risks while ensuring effective treatment. A modular search on MEDLINE/PubMed (National Library of Medicine, Bethesda, MD, USA) was conducted using combined keywords and subtopic-specific queries to identify relevant literature on orthodontics during pregnancy. Most studies were literature reviews, followed by various observational, survey-based, clinical, and case report designs. Orthodontic care during pregnancy must be adapted to trimester-related risks, with minimal interventions in the first trimester, elective procedures in the second, comfort-focused management in the third, and treatment of complex care postpartum. This protocol requires individual adaptation, highlighting the need for personalized medicine and coordinated input from multiple specialists to address orthodontic, obstetric, and psychological factors during pregnancy.

## 1. Introduction

Orthodontic treatment is viable during pregnancy, a period often viewed by patients as an opportune time to prioritize oral healthcare [1]. Physiologically, pregnancy does not constitute a contraindication to orthodontic treatment, provided that certain clinical and safety criteria are respected.

Pregnancy is characterized by transient, multisystemic physiological adjustments. These include cardiovascular, respiratory, hematological, gastrointestinal, renal, endocrine, and immune systems [2].

Changes also occur in the stomatognathic system. The etiology of these alterations lies in the hormonal profile, which fluctuates throughout pregnancy. The hormones estrogen and progesterone are the principal mediators of these oral cavity changes. Estrogen is a steroid hormone secreted by the ovaries and the placenta, and its most bioavailable form, estradiol, increases progressively, reaching peak levels of 7192 pg/mL in the third trimester, while progesterone ranges between 100 and 200 ng/mL. Estrogen increases tenfold and progesterone thirtyfold during pregnancy. The levels of these hormones also influence systemic inflammatory responses and bone metabolism [3].

The most observed adverse effects in the oral cavity caused by the hormonal profile typical of pregnancy include.

### 1.1. Gingival Hyperplasia, Gingivitis, and Periodontitis

Elevated estrogen levels increase capillary permeability, predisposing the patient to gingival hyperplasia and gingivitis. The interdental papilla and marginal gingiva are the areas most frequently affected by pregnancy gingivitis, which is often associated with pre-existing gingivitis. The absence of plaque and good oral hygiene can reduce its severity. If left untreated, gingivitis may progress to periodontitis [4].

Although pregnancy is not a direct cause of periodontitis, it can worsen pre-existing gingivitis, which could lead to periodontitis [5].

### 1.2. Pyogenic Granuloma

Increased angiogenesis caused by hormonal changes, combined with inflammatory factors such as plaque, may lead to a pyogenic granuloma. It appears in 1–5% of pregnancies and commonly develops on the labial aspect of the interdental papilla [6].

### 1.3. Caries Risk

Another common aspect of pregnancy is nausea and vomiting (hyperemesis gravidarum), caused by elevated gonadotropins during the first trimester.

Reduced salivary flow, causing xerostomia (increasing caries risk), diminishes the buffering capacity needed to counteract the acidity produced by reflux and vomiting [3]. Reflux, along with frequent vomiting, can negatively affect enamel mineralization, increasing the risk of dental erosion and carious lesions [7].

Gingival irritation may also make brushing and plaque removal uncomfortable or painful, accelerating caries development.

Hormonal changes affect not only salivary quantity but also its quality: levels of potassium ions, proteins, and salivary estrogen increase, while sodium ions and pH decrease [8].

## 2. Scope and Objectives

This study seeks to consolidate existing information into structured clinical recommendations—a practical framework serving as an accessible, chair-side reference across the three distinct clinical scenarios of pregnancy. The research focuses on gathering and synthesizing evidence from literature specifically concerning the orthodontic management of the gravid patient.

The primary aim of this study is to define and analyze safe and appropriate clinical recommendations during pregnancy for orthodontic treatment, with a specific focus on risk prevention and the management of potential complications. The overarching goal is to ensure maternal and foetal safety through targeted, preventative therapeutic planning that accounts for the physiological, psychological, and hormonal changes characteristic of pregnancy.

Specifically, this study aims to:

- Optimize treatment timing for various orthodontic procedures across the different gestational trimesters, optimizing the therapeutic workflow to prevent delays or relapses.
- Evaluate the relevance of pregnancy-specific conditions associated with invasive procedures—such as orthodontic extractions, the insertion of miniscrews (TADs), and the surgical–orthodontic exposure of impacted teeth—proposing selection criteria and safe management strategies.
- Assess psychological aspects, motivation, and patient compliance throughout the different trimesters, particularly regarding invasive clinical procedures.

By doing so, this study seeks to provide a linear and streamlined clinical pathway based on prevention, timing optimization, and the personalization of orthodontic care, intended to bolster both the safety and the efficacy of orthodontic treatment during pregnancy.

This research is intentionally designed as an overview of existing literature instead of a typical narrative review or systematic review. It seeks to connect theoretical research with everyday practice by synthesizing the essential clinical parameters found in the chosen articles, offering orthodontic practitioners a practical and readily available reference tool.

This protocol maintains a focus on systemically healthy pregnant patients to create an essential clinical baseline and to ensure clinical specificity. By focusing on the physiological and hormonal factors specific to a typical pregnancy, these clinical recommendations establish a standard framework that can subsequently be modified for complicated situations involving systemic comorbidities. These include intricate psychiatric or obstetric conditions that would require a degree of multidisciplinary knowledge, particularly in gynecology and clinical psychology, which exceeds the established epistemological limits of this orthodontic study. By maintaining this emphasis, this practical framework eliminates the danger of offering shallow advice on intricate systemic disorders.

## 3. Materials and Methods

This paper presents a clinical protocol based on a thematic overview rather than a conventional narrative review or meta-analysis. The objective is to synthesize clinical findings into an accessible, chair-side framework, prioritizing clinical applicability over exhaustive quantitative assessment.

The literature search utilized MEDLINE/PubMed (National Library of Medicine, Bethesda, MD, USA) and Google Scholar (Alphabet Inc., Mountain View, CA, USA), with supplementary queries in Scopus (Elsevier, Amsterdam, The Netherlands) and the Cochrane Library (John Wiley & Sons, Hoboken, NJ, USA). While the latter two databases were thoroughly scrutinized, they yielded no additional relevant studies that met the stringent clinical focus of this synthesis. The majority of studies were excluded due to confounding variables that deviated from the management of the systemically healthy patient; these included complex biological and genetic factors, systemic comorbidities such as gestational diabetes or obesity, and specialized craniofacial pathologies like cleft palate or orthognathic surgical cases. Furthermore, studies involving intrauterine dentistry, artificial intelligence, or pediatric demographics were omitted to preserve the protocol’s clinical specificity.

The choice for using PubMedis justified by the clinical nature of the study, which seeks to synthesize the current state of the field rather than perform an exhaustive meta-analysis of all existing data.

The decision to utilize a PubMed-centered search strategy is based on two key factors:

1. Medical Subject Headings (MeSH) provided a gold-standard controlled vocabulary. This ensured that the search results were clinically relevant and filtered for human-centered medical outcomes, which are often diluted in broader, multidisciplinary databases.
2. The core of orthodontic and obstetric literature is indexed within MEDLINE. By prioritizing this database, the study ensures that the clinical recommendations are derived from high-impact, peer-reviewed journals where major clinical breakthroughs and safety protocols are traditionally published.

### 3.1. Search Filters and Inclusion Criteria

The search was limited to literature published between 2000 and 2025 to ensure the inclusion of contemporary clinical advancements and safety protocols. Furthermore, to facilitate preliminary assessment of clinical relevance, only articles with available abstracts were included. Finally, to ensure a high level of comprehension and accuracy during the synthesis of guidelines, the search was restricted to articles published in English or Italian.

### 3.2. Exclusion Criteria

To ensure that the practical framework was based on high-quality, generalizable evidence, a rigorous set of exclusion criteria was established. This allowed for the removal of confounding variables, such as systemic comorbidities (e.g., gestational diabetes), complex craniofacial pathologies (e.g., cleft palate, orthognathic surgery), which fall outside the scope of routine orthodontic management during pregnancy.

In addition to the aforementioned filters, the following exclusion criteria were applied to refine the search results and ensure the clinical focus of the handbook:

- Study Type: Case reports, animal studies, and in vivo laboratory studies were excluded (except where specifically used as primary filters) to prioritize clinical evidence applicable to human patients.
- Thematic Exclusions: Titles and abstracts containing the following terms or topics were excluded to maintain the specific scope of this study:
  ○ Biological and Genetic: Molecular biology, histology, genetics, and in vitro fertilization.
  ○ Systemic and Comorbid Conditions: COVID-19, obesity, gestational diabetes, fetal alcohol syndrome, mental health disorders, and physical disabilities.
  ○ Craniofacial and Structural: Craniofacial syndromes or deformities, craniofacial growth and morphology, orthognathic surgery, maxillary sinus studies, and cephalometric values.
  ○ Complex Pathologies: Cleft lip and palate, obstructive sleep apnea (OSA), dental agenesis, bone grafts, and rare tumors.
  ○ Specialized Treatments: Root resorption, dental autotransplantation, gingival smile (gummy smile), botulinum toxin, and intrauterine dentistry.
  ○ Technology and Demographics: Pediatric dentistry (pedodontics), adolescent-only studies, teledentistry, and artificial intelligence (AI).
- Geographic and Population Bias: Studies limited to a single specific geographic area or restricted to narrow ethnic/demographic groups were excluded to ensure the global applicability of the proposed clinical protocol.

Given the interdisciplinary nature of the subject—encompassing orthodontics, maternal physiology, and pharmacology—a modular search strategy was employed, utilizing targeted Boolean queries to isolate specific clinical sub-themes.

The search modules were categorized as follows:

1. Core Clinical Intersections:
  ○ (orthodontics OR orthodontic treatment) AND (pregnancy OR pregnant patient)
  ○ pregnancy AND (dentistry OR dental OR oral health)
2. Pharmacology and Radiology Safety:
  ○ dental anesthesia AND pregnancy
  ○ (dentistry) AND (antibiotics) AND (pregnancy)
  ○ analgesics AND dental AND pregnancy
  ○ (dental) AND (radiology) AND (pregnancy)

This segmented approach ensured that every aspect of the clinical guidelines was supported by a dedicated body of evidence, covering radiological and pharmacological safety.

## 4. Results

The modular search strategy yielded a total of 1220 articles. Following initial screening of titles and abstracts and the application of predefined exclusion criteria—specifically the removal of case reports, non-human studies, and unrelated systemic pathologies—a final sample of 73 peer-reviewed articles was selected for full-text analysis.

These papers underwent a secondary qualitative screening. Analysis of the abstracts revealed that subscription-based articles primarily corroborated the physiological principles and safety protocols already established within open-access literature. Consequently, the inclusion of proprietary sources would have yielded redundant data without providing novel clinical insights or altering the proposed guidelines. By prioritizing high-quality, verifiable evidence, this study constructs a robust evidentiary foundation that serves as a practical framework for practitioners globally, irrespective of their resource environment. This overview aims to bridge the gap between theoretical research and pragmatic application and to provide a reproducible and chair-side reference for the orthodontic management of the gravid patient.

Following the synthesis of results from primary clinical searches and targeted psychological expansion, and the application of final criteria regarding free full-text accessibility and thematic relevance, 15 articles were definitively selected for inclusion.

A comprehensive breakdown of the study selection process, thematic focus, and classification of studies by research designs is presented in Table 1, Table 2 and Table 3.

## 5. Discussion

1. Clinical Assessment and Protocol for Orthodontic Treatment during Pregnancy

To evaluate clinical eligibility for orthodontic treatment during gestation, the clinician must conduct a comprehensive medical, dental, and obstetric history, encompassing prior reproductive outcomes, current gestational age, associated complications, and the use of pharmacological agents such as heparin, medications for gestational diabetes, or drugs for pregnancy-induced hypertension [9]. Direct consultation with the patient’s obstetrician is mandatory to exclude high-risk complications and assess the patient’s tolerance for local anesthesia and surgical stress [6]. Non-urgent procedures—such as canine exposure—are usually postponed until after delivery to avoid unnecessary risks; however, if the intervention is deemed necessary to prevent damage to adjacent incisors or cyst formation, the second trimester is the preferred time window [8]. Such procedures should only be performed if the patient is adequately motivated, understands the associated risks and benefits, and provides informed consent following obstetric approval. Regarding treatment eligibility, patients should initiate therapy only upon achieving periodontal stability and a caries-free status, characterized by the absence of plaque, calculus, soft tissue inflammation, and active carious lesions. For planned pregnancies, preemptive management and periodic dental evaluations are essential, while for unplanned pregnancies, the second trimester serves as the optimal window for restorative and endodontic interventions [8]. The patient’s motivation and attitude toward treatment must be monitored throughout gestation, as these may fluctuate alongside physical and psychological changes, and it remains the responsibility of the orthodontist and dental hygienist to provide targeted instructions for home care, such as the use of interdental aids, to maintain hygiene despite the presence of appliances [5]. Finally, therapeutic objectives must be clearly defined to prioritize conservative, non-invasive modalities, ensuring the patient is willing to adhere to meticulous hygiene and the defined treatment schedule [6].

2. Safe and Appropriate Orthodontic Treatment During Pregnancy

This clinical framework prioritizes the prevention of risks and the management of potential complications by integrating physiological and hormonal considerations directly into the therapeutic pathway. By adopting this streamlined approach, the study provides a linear clinical pathway centered on prevention, optimal timing, and personalized care, ultimately enhancing both the safety and efficacy of orthodontic treatment during pregnancy.

3. Proposed Orthodontic Treatment During Pregnancy

Three case scenarios are considered—one ideal (case I) and two exceptional (case II and case III)

**CASE I:** the ideal situation, in which a patient who, in the process of planning a pregnancy, requests an orthodontic evaluation and wishes to begin orthodontic treatment that will last throughout her gestation.

**CASE II:** an exceptional case in which the patient is already pregnant and wishes to start orthodontic treatment.

**CASE III:** an exceptional situation in which the patient becomes pregnant while orthodontic treatment is already underway.

4. Orthodontic Treatment Across the Trimester

### 5.1. Chronological Protocol: Ongoing and Planned Treatments (Case I and Case III)

#### 5.1.1. Case III

**Treatment Plan**

For a patient who becomes pregnant during orthodontic treatment, the same guidelines regarding the timing and planning are applicable as for a patient who begins treatment during a planned pregnancy, since the treatment plan had already been established before the pregnancy started (case I discussed below).

**Psychological Aspects**

Unlike cases I and II, this situation requires special attention to the mother’s psychological well-being, as her concerns and anxieties may impact treatment planning and compliance.

The patient may voice concerns regarding the potential contraindications of orthodontic treatment, particularly concerning maternal and fetal well-being. In such a case, effective communication on the orthodontist’s part is essential: it is their responsibility to provide clear and reassuring information, emphasizing that orthodontic treatment—when planned and executed with consideration for the patient’s pregnancy and all clinical and ergonomic changes—is both feasible and safe during pregnancy.

The following table (Table 4) summarizes this concept:

#### 5.1.2. Case I

**FIRST TRIMESTER**

The patient may have already begun orthodontic treatment or may be starting it (bracket placement).

The first trimester is the most critical period of pregnancy: organogenesis occurs and the fetus is susceptible to teratogenesis.

Elective procedures are deferred to the second trimester, while urgent conditions must be treated promptly, as their systemic risks outweigh fetal risks associated with necessary medications or anesthesia. Urgent problems must be managed at any stage of pregnancy [10].

In non-urgent cases, all procedures involving radiographs [11], antibiotics [12]/analgesics [13], or anesthesia [14]—such as orthodontic extractions, TAD insertion, and surgical exposure—should be avoided.

**Psychological Aspects and Cooperation**

During the first trimester, patients may experience nausea, vomiting, food aversions, fatigue, mood swings, and anxiety about the outcome of pregnancy. These factors may reduce compliance [15]:

- Missed or cancelled appointments due to morning sickness.
- Hygiene maneuvers (brushing, flossing) may trigger gag reflexes.
- Orthodontic discomfort may be harder to tolerate when combined with nausea.

**Logistical and Technical Considerations**

Depending on the type of orthodontic treatment (clear aligners or fixed appliances), it is possible to begin safely even in the first trimester, as anesthesia or analgesics are typically unnecessary in the initial phase. Light archwires and moderate forces are sufficient. Adjustment appointments are usually scheduled monthly.

Preventive care becomes essential, as pregnancy combined with fixed appliances significantly increases the risk of gingivitis and cavities. Communication plays a key role in maintaining compliance and reinforcing preventive habits, especially hygiene with orthodontic appliances.

The following points synthesize the primary clinical considerations discussed above [3]:

- Use light orthodontic forces to reduce discomfort.
- Offer flexible scheduling, avoiding early-morning appointments.
- Keep visits short.
- Reinforce hygiene instructions and suggest alternatives (soft-bristle brushes, mouth rinses)
- Reassure the patient and provide clear information to reduce anxiety.
- Create a comfortable, relaxing environment.

**SECOND TRIMESTER**

The second trimester is the safest period for dental procedures requiring anesthesia because:

- Fetal organogenesis is complete.
- Patient positioning is still comfortable.
- Any complications can be pharmacologically managed.
- Teratogenesis risk is reduced/absent.

**Psychological Aspects and Cooperation**

Patients typically have higher energy, nausea resolves, and emotional stability increases.

Compliance is usually optimal. The patient can attend visits regularly and tolerate longer sessions [15].

**Logistical and Technical Considerations**

If the treatment plan includes orthodontic extractions, canine exposure, or TAD placement, this is the most appropriate phase for performing them.

Even simple procedures (e.g., extraction) may produce unexpected intraoperative complications (e.g., root fracture), potentially increasing patient anxiety.

Therefore:

- Treatment should proceed only after thorough informed consent, with the patient aware of all risks.
- Psychological stability is required; anxiety may cause hypertension and tachycardia.
- Obstetric approval is mandatory.
- The dentist must evaluate the patient’s psychological state on the day of the procedure.

If the patient expresses anxiety or hesitation, the procedure should be postponed.

More complex interventions (e.g., difficult extractions, canine exposure) should:

- Be performed by an oral surgeon.
- Include anesthetic monitoring and vital sign assessment.
- Implement a surgical guide fabricated from a CBCT taken at the beginning of treatment to minimize risks and simplify the procedure.

**THIRD TRIMESTER**

**Effects on Orthodontic Compliance**

During this period, the patient may experience fatigue, back pain, breathing difficulty in the supine position, urinary frequency, sleep disturbances, and increased anxiety about the upcoming birth. These factors may reduce compliance, which may manifest as:

- Missed appointments due to discomfort.
- Orthodontic discomfort becomes harder to tolerate.
- Decreased use of removable orthodontic appliances.
- Swollen, sensitive gums make hygiene more difficult.
- Attention is often focused on childbirth.
- Some patients may request treatment suspension until after delivery.

**Strategies to Improve Compliance**

1. Ensuring patient comfort becomes essential:
  - Avoid prolonged supine positioning to prevent vena cava compression.
  - Reposition the patient every 3–7 min.
  - Place a cushion under the right side of the abdomen [8].
2. To optimize treatment during this trimester:
  - Schedule short, comfort-oriented appointments.
  - Choose times of day when the patient feels more rested.
  - Temporarily reduce orthodontic force intensity
  - Avoid painful or complex maneuvers (tight activations, new rigid wires, major tooth movements)
  - Postpone demanding phases (space closure, complex biomechanics) until postpartum.

The primary objective is to maintain safe and tolerable continuity of treatment without increasing physical or psychological discomfort.

**IMMEDIATE POSTPARTUM**

The postpartum period is ideal for performing deferred radiographic evaluations and completing orthodontic procedures that were contraindicated or postponed during pregnancy. Managing complications is easier pharmacologically and surgically than during pregnancy.

Orthodontic treatment may resume according to the original plan or a revised one if appointments were suspended.

**Motivation and Compliance After Delivery**

Patient motivation varies depending on the postpartum phase and maternal responsibilities.

- **First 3–6 months:** fatigue, newborn care, and lifestyle adjustments could lead to reduced motivation, leading to lower compliance, missed appointments, poor oral hygiene, and inconsistent orthodontic appliance use. Appointment scheduling must be flexible.
- **After 6 months:** routines stabilize, motivation increases, and orthodontics may be seen as part of self-care and identity recovery. At this stage, emphasizing aesthetic benefits and proposing clear aligners (if compatible with the treatment plan) can enhance motivation and improve self-esteem.

The following table (Table 5) provides a summary of the proposed clinical protocol, correlating gestational phases with specific therapeutic priorities and psychosocial profiles for the ideal case (case I).

### 5.2. Strategic Management of New Patients (Case II)

#### Case II

**Treatment Plan**

In a pregnant patient with no particular orthodontic treatment demands other than alignment of the anterior teeth, and who has a panoramic radiograph taken within the last two years, orthodontic treatment may be initiated, provided that the orthodontist performs an accurate case evaluation based on the analysis of intraoral and extraoral photographs, as well as the study of dental models. This approach allows safe and appropriate treatment planning, even in the absence of recent radiographic updates, while ensuring proper assessment of dental relationships and dentofacial morphology.

In the presence of a skeletal discrepancy requiring orthognathic surgery, there are two options:

If the patient wishes to undergo orthodontic–surgical treatment, it is suitable to postpone starting the treatment plan until after pregnancy. This is because orthognathic surgery requires radiographic imaging such as lateral cephalometry, anteroposterior cephalometry (in cases of asymmetry), and CBCT in addition to a panoramic radiograph, as well as a multi-step treatment plan involving dental extractions, pre-surgical orthodontics, reassessments, and surgical preparation, which may itself require further radiographic investigations. As this is not a condition that requires urgent treatment, radiation exposure during any trimester of pregnancy would not be justified.

If the patient has a panoramic radiograph taken within the last two years, a limited orthodontic treatment aimed at aligning the teeth with orthodontic camouflage of the skeletal discrepancy may be considered. The informed must clearly state that the patient has been fully informed of these compromises and accepts them knowingly.

The clinical management of new patients necessitates a careful evaluation of treatment complexity and diagnostic needs to ensure maternal and fetal safety. The following table (Table 6) outlines the strategic treatment planning for new orthodontic cases during pregnancy, distinguishing between those requiring simple alignment and those presenting with skeletal discrepancies.

5. Timing of treatment initiation by modality and trimester

If treatment is carried out using aligners, it may be started in any trimester.

When treatment with a traditional fixed appliance is chosen, special attention must be paid during the third trimester—and possibly toward the end of the second trimester. The placement of orthodontic brackets can be lengthy, lasting 30–40 min. During this procedure, it is important to avoid keeping the patient in a supine position for extended periods; if maintaining this position is necessary, it is recommended to adjust her posture every 3–7 min and schedule breaks to ensure comfort and safety during pregnancy.

The clinical selection between various orthodontic modalities, specifically contrasting the procedural demands of fixed appliances with the flexibility of clear aligners throughout the gestational period, is summarized in Table 7.

## 6. Integration of Psychosocial Factors

A primary methodological challenge identified during this review concerns the psychosocial dimensions of the gravid patient, as no existing literature directly correlates orthodontic intervention with gestational psychological transitions. While general psychological studies exist, they were excluded due to the presence of confounding variables such as pre-existing psychiatric comorbidities. Consequently, the psychological framework of this protocol was constructed through the theoretical extrapolation of data from a single pivotal study regarding general gestational behavioral shifts. Given the complete absence of pregnancy and orthodontics-specific psychological literature, the behavioral and motivational recommendations provided herein represent an original application of general gestational psychology to the dental setting. This synthesis established a necessary clinical baseline where none previously existed, highlighting an undeniable imperative for future interdisciplinary research to investigate the reciprocal influence between malocclusion management and maternal well-being.

It must be noted that several procedural recommendations within the discussion—particularly those concerning immediate patient ergonomics and chair-side communication—are not derived from dedicated clinical trials, but rather from logical clinical corollaries. In the absence of primary research, these guidelines rely on expert consensus and the pragmatic application of safety standards to mitigate the unique physiological demands of the gravid state.

## 7. Limitations

This overview is subject to several critical limitations that warrant acknowledgement. First, the exclusion of subscription-based literature represents a definitive boundary; by omitting articles behind paywalls, this study may lack certain proprietary data or specific case reports found in closed-access journals (the rationale for which is deferred to Section 4). Second, the scope is strictly confined to the systemically healthy patient, which constitutes a major clinical limitation. It does not account for the management of prevalent gestational comorbidities—such as diabetes, hypertensive disorders, or pre-existing psychiatric conditions—rendering this protocol inapplicable to high-risk obstetric populations. Furthermore, the reliance on PubMed/MEDLINE as the primary source means that niche or localized studies indexed in other specialized databases were likely overlooked. Finally, the most significant limitation is the absolute lack of primary research regarding the psychological profile of the orthodontic patient during pregnancy. Because no specific literature exists, the behavioral guidelines provided here are merely a theoretical application of general psychology, rather than evidence-based orthodontic data. These gaps highlight that while this framework provides a baseline, it remains incomplete regarding complex medical cases and direct psychosocial evidence.

## 8. Conclusions

In summary, this overview establishes a foundational clinical protocol for the orthodontic management of the gravid patient, emphasizing the necessity of a personalized approach. The synthesized evidence suggests that while orthodontic intervention is fundamentally safe, its efficacy depends on a profound sensitivity to the patient’s evolving physiological and psychological state.

While not all clinical permutations have been fully explored, this study provides a foundational orthodontic protocol for the management of the gravid patient. It is crucial to highlight that the suggestions outlined here are specifically tailored for the systemically healthy individual. Pregnancy often interacts with intricate systemic comorbidities—like gestational diabetes, hypertensive issues, and prior psychiatric disorders—which inherently modify physiological and hormonal reactions. Managing these complex cases necessitates highly personalized, multidisciplinary medical supervision that extends beyond the limits of a standard orthodontic framework. As a result, there is a strong need for additional studies to tackle these particular clinical intersections. Future interdisciplinary research combining orthodontics, gynecology, and clinical psychology is crucial for developing specific, evidence-driven sub-protocols designed for high-risk obstetric groups. In the end, although this framework acts as a dependable basis for standard care, the effective handling of the pregnant patient will consistently rely on personalized, patient-focused clinical judgment.

Furthermore, this study identifies a significant lacuna in the current literature regarding the emotional and cognitive experience of the orthodontic patient during gestation. There is a compelling need for future prospective research wherein the psychological aspects of pregnancy and orthodontic treatment are studied simultaneously. Investigating the reciprocal influence between orthodontic discomfort and gestational mood fluctuations is essential to transition from a purely technical protocol to a holistic model of maternal care.

## Figures and Tables

**Table 1:** Refining study selection and yield.

Criteria Applied	Result
Filters (2000–2025, Abstract, EN/IT)	1.221
Inclusion/Exclusion Criteria	−1.148
Qualitative review for relevance	−59
Articles included	14
Psychological aspects of pregnancy	1

**Table 2:** Number of articles by subtopic.

Subtopic	Number of Articles
General Dentistry and Pregnancy	4
Orthodontics and Pregnancy	6
Pharmacology	2
Radiology	1
Anesthesia	1
Psychological aspects	1

**Table 3:** Distribution of studies by design.

Study Type	Number of Articles
Review	13
Systematic Review	1
Qualitative analysis	1

**Table 4:** Clinical Management of Ongoing Orthodontic Treatment (Case III).

Scenario	Treatment Strategy	Diagnostic and Imaging Constraints
Case III: Pregnancy During Treatment	Maintain established plan but adapt ergonomics/timing to the current trimester Focus on patient reassurance.	Adhere to the same safety/deferral guidelines as Case I for any mid-treatment changes.

**Table 5:** Comprehensive Orthodontic Management Protocol: Clinical and Ergonomic Guidelines by Gestational Trimester.

Phase	Primary Clinical Objective	Psychosocial and Compliance Profile	Key Technical Protocol
*First Trimester* (Critical Phase)	*Stasis and Risk Mitigation:* Defer elective procedures; management of emergencies only.	High malaise; heightened gag reflex and nausea may impede hygiene and attendance.	Short, afternoon appointments; minimize appliance activation; avoid all pharmacological/radiographic stressors.
*Second Trimester* (Optimal Window)	*Active Intervention*: Ideal period for necessary extractions, TADs, or surgical exposures.	Peak emotional stability and physical energy; optimal tolerance for longer sessions.	Obtain formal obstetric clearance; monitor vital signs; assess surgical anxiety prior to intervention.
*Third Trimester* (Comfort Phase)	*Maintenance and Safety*: Ensure continuity while preventing maternal physical distress.	Increasing fatigue and “nesting” focus; potential decline in oral hygiene consistency.	Semi-upright positioning; use right-side cushions to prevent Vena Cava Compression; defer complex biomechanics.
*Postpartum* (Recovery Phase)	*Resumption and Finalization*: Integration of deferred diagnostics (radiographs) and complex goals.	Initial high fatigue (0–6 months) transitioning to stabilized motivation (6+ months).	Flexible scheduling; prioritize aesthetic outcomes (e.g., clear aligners) to reinforce patient self-esteem.

**Table 6:** Strategic Treatment Planning for New Orthodontic Cases during Pregnancy (Case II).

Scenario	Treatment Strategy	Diagnostic and Imaging Constraints
Case II: New Patient (Alignment Only)	Can initiate treatment if a panoramic X-ray from the last 2 years is available Focus on intra/extraoral photos and study models.	No new radiographs justified for simple alignment during pregnancy.
Case II: New Patient (Skeletal Discrepancy)	Option A: Postpone surgical-orthodontic plans until postpartum (requires heavy radiation: CBCT, Ceph) Option B: Orthodontic camouflage (aligning teeth only).	Informed consent must explicitly state the “compromise” of camouflage treatment vs. surgery.

**Table 7:** Differential Management of Orthodontic Modalities: Fixed Appliances vs. Clear Aligners across Clinical Scenarios.

**Appliance Specifics (All Cases)**	**Aligners:** Can be started in any trimester **Fixed Appliances:** Caution in 3rd Trimester due to long bonding sessions (30–40 min)	**Ergonomic Rule:** Frequent breaks and postural adjustments during long bonding procedures

## Data Availability

No new data were created or analyzed in this study. Data sharing is not applicable to this article.
